# Resetting and Transplanting Teeth

**Published:** 1876-12

**Authors:** H. N. Wadsworth

**Affiliations:** Washington, D. C.


### ARTICLE III.

### *Resetting and Transplanting Teeth.*

BY H. N. WADSWORTH, D. D. S., WASHINGTON, D. C.

Hundreds and thousands of patients of intelligent, discriminating, appreciative minds and plethoric purses, who would gratefully accept almost any alternative rather than resort to an artificial tooth, are yet doomed to do so in cases quite unnecessary because unaware of a remedy; because many of us as a profession have not yet seized hold of and applied a principle that nature is constantly illustrating for us, that every little while the country clown is practically and successfully applying for our imitation.

Look back on any of our mental records, and call to mind the number of teeth and roots that have lost their original position ; that are at the time we remove them entirely changed from their original location ; that are to all intents and purposes dead internally and externally, nerves and blood-vessels, some even lying at right angles with their former position, *i. e.* flat on the gum, and yet, when we examine them, evincing a remarkable evidence of healthy action, and requiring a considerable amount of lancing to free them from their attachment to the gum, and of force to remove them.

We see a country clown who has received a kick from a horse or mule, a blow from any of the thousand and one sources from which they may emanate, and a front tooth is knocked out. It is picked up, pushed into its place, and with no attention whatever returns to its duty and becomes a valuable and useful member.

With these useful practical hints of what nature is doing to correct the ravages of disease, of what uneducated boors can do to remedy the disfiguring results of a painful accident, what are *we* doing as an educated body of scientific men to meet these forlorn hopes in our practice ; in applying these principles that nature on the one hand and ignorance on the other have thrust before us, to awaken our dormant ideas and shame our boasted skill as professional men?

Alveolar abscesses of the four inferior incisors, engendered ed by calculus and allowed to run its destructive course is one of the crying evils of our profession; one of the most difficult diseases to contend against; one the least skillfully handled, receiving the least attention and resulting in the most unfortunate deprivation that can occur from the simple loss of one or more teeth. Unfortunately, the hurried, inefficient manner in which the first symptoms are treated by many in our profession, added to the difficulty of impressing on our patients the necessity of repeated and constant care not only on their part, but also on the part of their operator allows the disease to acquire such headway as to render all the usual remedies unavailing, and a loss of these teeth, one, two, or all, an inevitable necessity.

Having arrived at that condition of disease when no efforts of skill will restore a tooth to a healthy condition, we proceed in the first place to destroy (if alive) the nerve and to remove it, filling afterwards with gold. In most cases of abscess the nerve will be found already dead, and no particular trouble will be necessary in approaching and removing it. This we consider a necessary preliminary step, otherwise the sulphur evolved by decomposition of the nerve will permeate the tubuli of the bone, depositing its darkening, discoloring product under the enamel, giving rise to the opaque, disfiguring expression so disagreeable and so objectionable. This entire operation should be done previous to extraction, as the less the tooth is handled before being replaced the more probability of a successful result.

Having brought the tooth into as healthy condition internally as can be done, we arrive at the time for its extraction, previous to which we should make suitable arrangements for securing it *firmly* in position for a sufficient length of time to enable it to become once more solidly established in its former position; for this purpose, with a piece of the usual sheet gutta-percha of double thickness, fit a cap accurately over the offending tooth, and over one, two, or three on each side of it, as may appear necessary, so as to give the splint a firm and unyielding basis to prevent any possibility of a movement of the tooth after it is replaced, and that it may be forced firmly into and be steadily retained in its socket. This retaining of the tooth *in absolute repose* is of the utmost consequence,—quite as much so as the retention of any broken bone that it may "knit," and if not carefully and thoroughly insisted upon the whole process will probably result in a failure. That the cap or splint may have sufficient durability, it is a good plan to add a narrow strip along its cutting edge to insure its withstanding the antagonizing action of mastication.

Having completed the preliminary arrangements, extract the tooth; carefully and gently remove all traces of the abscess sac, if one existed, and all calculi, but do not touch or remove any healthy periosteum that may perhaps still exist, for in this healthy portion left, we hope exists a nucleus for more and for a healthy returned vitality. The disease and the cause of the disease having been removed, we may fairly anticipate a return to comparative health and a considerable amount of comfort and usfulness.

The tooth being prepared with all the rapidity possible, take an instrument and fix a cone of soft Japanese paper on the end, with which carefully and gently wipe out the socket, to remove any fibers of the abscess sac that may remain; wash out the socket by injections of warm water; then press the tooth (after rinsing it in warm water) firmly into its former position, holding it for a few moments with the fingers, and rinsing the mouth until the blood has nearly ceased to flow; then carefully adjust the cap or splint, which a skillful operator can adjust so as to be a tight, snug fit, and holding firmly to its position without danger of coming off or loosening.

It is a good plan to let the patient wear this splint for two or three days (before extraction of the tooth,) seeing it daily, and trimming the edges so as to have it free from any irritation to the gum before actual application to the reset tooth. A wash should be used three or four times daily and the mouth kept thoroughly clean with the brush, but on no account should the splint be removed or disturbed for at least ten days; then its removal for the purpose of examination should be carefully done as follows: with a sharp knife cut a flap from the edge of the gutta percha splint down on each side of the tooth to the gum; bend this flap over and away from the tooth, so as to entirely loosen it from its embrace around the tooth; if this does not entirely loosen its hold of the tooth, cut a similar flap from the inside of the splint and turn that down also, but do not in either case break the flap off; this will relieve the weak tooth from any danger of disturbance in removing the splint, which can now be done, and the tooth examined thoroughly, cleansed, and gently brushed. If not sufficiently "set," thoroughly dry the splint, carefully warm the flap or flaps, and gently attach them, and whilst warm readjust the splint and carefully press the warm flaps gently around the tooth and leave it a week longer, and then examine it again. If not fully set, try another week, when it will be found a success, probably, if it is destined to succeed. For a wash, use—

℞.—Tinct. myrrhæ, ℥ii;

Oleum gaultheriæ, ʒi;

Tinct. arnicæ, ʒi;

Acid. carbolici, gtt. xxx.

With this wash use a small quantity in water; let the patient cleanse the mouth frequently each day.

Put the patient on a nutritious but *unstimulating* diet; forbid positively all alcoholic stimulants of any kind, even beer; and last, but not least, tell the patient if your rules and directions are not zealously carried out not to hope for success, for a tooth in this condition is in the last stages, as one might say, of "typhoid fever," in which the chances are against recovery, unless under the most skillful nursing and the high courage of the patient.

Having disposed of one branch of our subject, we come to another and much more difficult one, requiring a much higher order of talent for success, the utmost skill and watchfulness in its manipulations, and far greater honor and reward in its success. Let us examine and inquire into the peculiar surroundings of "transplanting." First, what are the essentials of success? In the vegetable kingdom, in selecting a tree we choose a *young* and healthy one. We remove it from its location with the utmost care, deprive it of a good portion of its foliage, endeavor to save as many of its roots as possible uninjured, and those that are injured trim smooth with a sharp knife; the hole for its new position is large and roomy, the soil rich and loose, and nearly similar to that it occupied before; it is planted with the greatest care, watered frequently, tied up, and kept from moving about; the time of the day and time of the year are studied and carefully observed, and regulate the operation; and thus skillfully performed it is successful, if a thorough attention is extended to it for some time afterwards.

In choosing a tooth for transplanting, it should be from the mouth of a young and healthy person,—thoroughly healthy, lest we transplant also a dangerous subtle disease,—of the same temperament, if possible we may avoid having to reduce the size or change the shape of the tooth to have it fit. I look upon any cutting, or even scratching or bruising, as so many wounds to injure and render less certain the result; and every portion of periosteum remaining on the tooth should be carefully encouraged to remain, as it is of vital importance.

Having chosen the tooth we think corresponds in size, color, location, and all the other surrounding characteristics, to replace the one we intend to extract, carefully kill (if alive) the nerve and remove it, filling thoroughly to the apex, and completing the operation thoroughly. The tooth now being ready for removal, we proceed to prepare a gutta-percha splint, as described before; only, after it has become set and cold, we cut out carefully from the splint where the new tooth is to be located a sufficient amount to give it full room without touching, and this cavity in the splint is to have a small portion of warm gutta percha put around it as a lining when the new tooth is in position, and the splint is to be carried rapidly to its proper position.

If the tooth is not accurately calculated to enter the socket of the old one, it will be necessary to reduce it so as to have as good a "fit" as posssible, and every requisite to a rapid and skillful reduction of size should be at hand. A pair of callipers will be found useful in gauging its diameter, whilst the length can be easily adjusted by the old tooth placed by its side.

If not absolutely necessary, I would not change the external surface of the tooth in any particular whatever. I

would not wipe a drop of blood from its surface, but *instantly* insert it in its new location and at once apply the splint after the blood has ceased to flow.

All simple wounds heal by first intention, if the air is not allowed to enter and cause oxidation. Make a cut in your hand and instantly press the lips of the wound together, holding them under water till a bandage is prepared ; then placing a wet cloth over the wound, bind it with the lips firmly together ; keep the cloth wet and the cut will close in an hour, and there will be no suppuration afterwards. Now it is equally important that we should transfer the new tooth instantly to its new location, if its size will admit not changing it in any particular, or even washing it ; the chances of success are far greater. If, however, it requires "trimming," then do it as quickly and carefully as possible and insert it ; and having washed off the blood with the mixture of the formula previously given, apply the splint.

It is still more necessary to keep the patient under unstimulating diet in these cases than in those of resetting, and it is well worth our utmost efforts to let no step be untaken to insure success. The removal of the splint after ten days should be done in the most delicate manner, as described before, and after washing the parts warm the flaps, carefully carry the splint to its proper position, and press the flaps nicely around the tooth and fasten them.

While it is always best to have a sound and healthy tooth for our purpose, one that has lost its vitality without loss of color, or even one that has had no abscess, but is decayed, can be used with success, provided the decay does not disfigure, the periosteum is healthy, and the color uninjured. —*Dental Cosmos.*

